# Treatment of Large-Diameter Truncal Veins: A Structured Review of Contemporary Endovenous and Surgical Modalities

**DOI:** 10.3390/jcm15145686

**Published:** 2026-07-20

**Authors:** Abhay Setia, Claus-Georg Schmedt

**Affiliations:** 1 Department of Vascular Surgery, DIAK Klinikum Landkreis Schwaebisch Hall, 74523 Schwaebisch Hall, Germany; 2 Department of Vascular Medicine, Division of Vascular and Endovascular Surgery, Klinikum Darmstadt, 64283 Darmstadt, Germany

**Keywords:** large-diameter truncal veins, great saphenous vein, endovenous thermal ablation, endovenous laser ablation, radiofrequency ablation, cyanoacrylate closure, mechanochemical ablation, ultrasound-guided foam sclerotherapy, high ligation and stripping

## Abstract

Truncal vein insufficiency can be treated with endovenous thermal ablation (EVTA), non-thermal non-tumescent techniques, ultrasound-guided foam or microfoam sclerotherapy, and high ligation with stripping. However, the optimal management of large-diameter truncal veins remains unclear. This structured review summarizes treatment outcomes in large-diameter incompetent truncal veins and compares available modalities according to vein diameter, anatomical success, recurrence, reintervention, and complications. A PubMed/MEDLINE search was performed for studies published between January 2000 and June 2026. Studies were eligible if they specifically evaluated incompetent great saphenous veins, small saphenous veins, or accessory saphenous veins described as large-diameter, large-caliber, very large, enlarged, or wide-diameter truncal veins. Twenty-three primary studies met the inclusion criteria. Definitions of large-diameter veins varied widely, ranging from >8 mm to >20 mm. EVTA represented the largest evidence base and generally showed high anatomical success, with most studies reporting occlusion, recurrence-free, or recanalization-free rates above 90%. Foam-based treatment, cyanoacrylate closure, and mechanochemical ablation were supported by fewer large-vein-specific studies, and durability in larger veins remains less certain. High ligation and stripping showed low recurrence or residual stump rates in selected comparative studies, but outcome definitions differed from endovenous occlusion endpoints. Current evidence supports individualized treatment selection based on vein diameter, anatomy, tortuosity, reflux pattern, and expected durability. Dedicated randomized trials using standardized diameter definitions and outcome reporting are needed.

## 1. Introduction

Truncal vein insufficiency is a common cause of symptomatic chronic venous disease and is treated by eliminating axial reflux. Available treatment modalities include endovenous thermal ablation (EVTA), such as endovenous laser ablation (EVLA) and radiofrequency ablation (RFA), non-thermal non-tumescent techniques including cyanoacrylate closure (CAC) and mechanochemical ablation (MOCA), ultrasound-guided foam or microfoam sclerotherapy, and conventional high ligation and stripping (HL/S) [1,2].

Over the last two decades, endovenous techniques have largely replaced conventional surgery as first-line treatment for truncal reflux because of their minimally invasive nature, high anatomical success, reduced postoperative morbidity, and faster recovery [1]. However, treatment outcomes may be influenced by anatomical factors, including vein diameter, tortuosity, reflux pattern, junctional anatomy, and the length of the incompetent segment.

Large-diameter truncal veins represent a clinically relevant subgroup in which optimal treatment selection remains less clearly defined. Larger vein diameter may reduce vein wall apposition, affect the distribution of thermal energy, sclerosant, or adhesive within the vein lumen, and influence the risk of incomplete closure, recanalization, recurrence, or thrombus extension [2,3]. The 2022 European Society for Vascular Surgery (ESVS) guidelines consider truncal veins >12 mm as large-diameter veins, although published studies have used heterogeneous thresholds ranging from >8 mm to >20 mm [1,2,3].

Despite the increasing use of endovenous and non-thermal techniques, evidence specifically addressing the treatment of large-diameter truncal veins remains scattered across different modalities, study designs, and diameter definitions. The aim of this review was therefore to summarize the available evidence on treatment outcomes in large-diameter truncal veins and to compare treatment modalities with regard to vein diameter, anatomical success, recurrence, reintervention, and complications.

## 2. Materials and Methods

### 2.1. Literature Search and Eligibility Criteria

A comprehensive literature review was conducted to identify studies evaluating the treatment of large-diameter truncal veins. The PubMed/MEDLINE database was searched for studies published between January 2000 and June 2026 using the following terms, either individually or in combination: “large diameter saphenous vein”, “large great saphenous vein”, “large saphenous vein AND EVLA”, “large saphenous vein AND radiofrequency ablation”, “large saphenous vein AND cyanoacrylate”, “large saphenous vein AND mechanochemical ablation”, “large saphenous vein AND stripping”, and “large diameter truncal vein”. An additional broad search using the terms “saphenous vein AND 12 mm” and “saphenous vein AND 10 mm” was performed to identify studies that used diameter-based inclusion criteria without explicitly referring to large-diameter veins.

**Figure 1:**
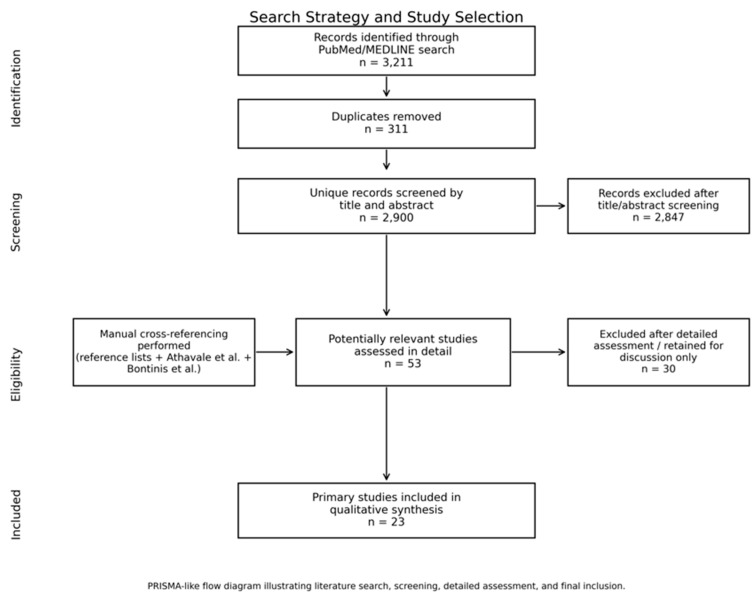
Search strategy and study selection flow diagram. Flow diagram showing record identification, duplicate removal, title/abstract screening, detailed assessment, cross-referencing [2,3], and final inclusion of primary studies in the qualitative synthesis.

Reference lists of relevant publications were manually reviewed. In addition, the systematic reviews by Athavale et al. and Bontinis et al. were used for cross-referencing and the identification of further potentially eligible studies [2,3] Figure 1.

### 2.2. Study Selection and Exclusion Criteria

The initial PubMed/MEDLINE search yielded 3211 records. After the removal of duplicates, 2900 unique articles underwent title and abstract screening. Potentially relevant articles were reviewed in detail according to the predefined eligibility criteria. Following detailed assessment and cross-referencing of relevant reviews, 23 primary studies met the inclusion criteria and were included in the qualitative synthesis. A total of 30 potentially relevant studies were excluded from the primary synthesis or retained only for discussion.

Studies were eligible if they specifically investigated treatment outcomes in incompetent great saphenous veins (GSV), small saphenous veins (SSV), or accessory saphenous veins that were explicitly described by the authors as large-diameter, large-caliber, very large, enlarged, or wide-diameter truncal veins, or if treatment of large veins constituted the primary objective of the study. Thermal ablation techniques, including EVLA and RFA, non-thermal techniques, including ultrasound-guided foam sclerotherapy (UGFS), microfoam ablation, MOCA, and CAC, and conventional HL/S were considered.

Where multiple publications described the same patient cohort, the study with the longest follow-up or the most comprehensive outcome reporting was selected. Studies evaluating unselected populations with truncal venous insufficiency without a predefined focus on large-diameter veins were excluded, even when vein diameter was reported as a secondary predictor of treatment outcome. Reviews, editorials, case reports, technical notes without clinical outcome data, animal studies, studies involving venous bypass grafts, studies addressing non-truncal venous pathology, and studies of treatment modalities outside the predefined scope were excluded.

Studies reporting treatment of localized aneurysmal changes or focal saphenous vein aneurysms without evaluation of diffuse large-diameter truncal venous incompetence were excluded from the analysis. Saphenous vein aneurysms should be distinguished from diffusely enlarged refluxing truncal veins. A venous aneurysm of a saphenous trunk has been defined as a focal dilatation of at least three times the upper limit of the average diameter, or as a diameter > 20 mm near the saphenofemoral junction for the GSV and >15 mm near the saphenopopliteal junction for the SSV [1].

### 2.3. Data Extraction and Evidence Synthesis

Data extraction focused on study design, patient and vein characteristics, definition of large-diameter veins, treatment modality, follow-up duration, anatomical success, occlusion, recanalization, recurrence, reintervention, and treatment-related complications. Extracted complications included deep vein thrombosis (DVT), endothermal heat-induced thrombosis (EHIT), thrombus extension, pulmonary embolism, and other reported adverse events. The data was tabulated in Microsoft Excel (Microsoft Corporation, Redmond, WA, Unites States of America).

Owing to substantial heterogeneity in study design, vein diameter definitions, anatomical measurement sites, treatment protocols, follow-up duration, and outcome reporting, a quantitative meta-analysis was not performed. The evidence was synthesized narratively and grouped according to treatment modality: EVTA, UGFS/microfoam ablation, CAC, MOCA, and HL/S.

### 2.4. Study Classification and Risk-of-Bias Assessment

Included primary studies were classified according to study design, including comparative observational studies, prospective observational studies, retrospective observational studies, registry/database studies, and case series. This classification was used to describe the overall level of evidence available for the treatment of large-diameter truncal veins.

Given the heterogeneity of the available literature and the narrative objective of this review, a formal risk-of-bias assessment was not performed. The review was conducted as a structured comprehensive review rather than a systematic review or meta-analysis.

### 2.5. Definition of Large-Diameter Veins

No universally accepted definition of a large-diameter truncal vein exists. The 2022 ESVS Clinical Practice Guidelines consider truncal veins > 12 mm in diameter as large-diameter veins [1]. Consequently, studies were included according to the definitions used by the original investigators. Across the included studies, definitions of large-diameter truncal veins were heterogeneous, with reported thresholds ranging from >8 mm to >20 mm depending on study design, treatment modality, anatomical measurement site, and investigator definition.

## 3. Results

### 3.1. Evidence Level of Included Studies

The literature search identified 3211 records. After the removal of duplicates, 2900 unique records underwent title and abstract screening. Following detailed assessment and manual cross-referencing, 23 primary studies met the predefined inclusion criteria and were included in the qualitative synthesis. The included evidence consisted of seven comparative treatment or protocol studies, ten diameter-comparison observational cohort studies, one prospective observational study, four observational case series or technical series, and one registry/database study. No randomized controlled trial specifically designed to evaluate treatment outcomes in large-diameter truncal veins fulfilled the final inclusion criteria (Table 1).

Of the 23 included primary studies, 21 included an EVTA arm or evaluated EVTA outcomes. Two studies evaluated foam-based truncal treatment using UGFS or microfoam ablation, two studies evaluated CAC, one study evaluated MOCA, and four studies included HL/S or a stripping-based surgical component. Because several comparative studies evaluated more than one treatment modality, modality counts are not mutually exclusive and do not sum to the total number of included studies. Follow-up duration ranged from 3 weeks to 48 months (Table 2).

### 3.2. Definitions of Large-Diameter Truncal Veins

No standardized definition of a large-diameter truncal vein was identified among the included studies. Reported diameter thresholds varied substantially, ranging from >8 mm to >20 mm, depending on the study design, treatment modality, anatomical measurement site, and original investigator definition. Florescu et al. further distinguished between large veins measuring 10–20 mm and very large veins measuring >20 mm [4].

### 3.3. Endovenous Thermal Ablation

Twenty-one primary studies included an EVTA arm or evaluated EVTA outcomes in large-diameter truncal veins (Table 3). These studies included EVLA, RFA, comparative EVLA/RFA studies, EVTA versus non-EVTA comparator studies, and one registry/database study [4,5,6,7,8,9,10,11,12,13,14,15,16,17,18,19,20,21,22,23,24]. Definitions of large-diameter veins ranged from >8 mm to >20 mm, and follow-up duration ranged from 3 weeks to 48 months. Most studies evaluated large-diameter GSVs, while selected studies also included SSVs or accessory saphenous veins.

**Figure 2:**
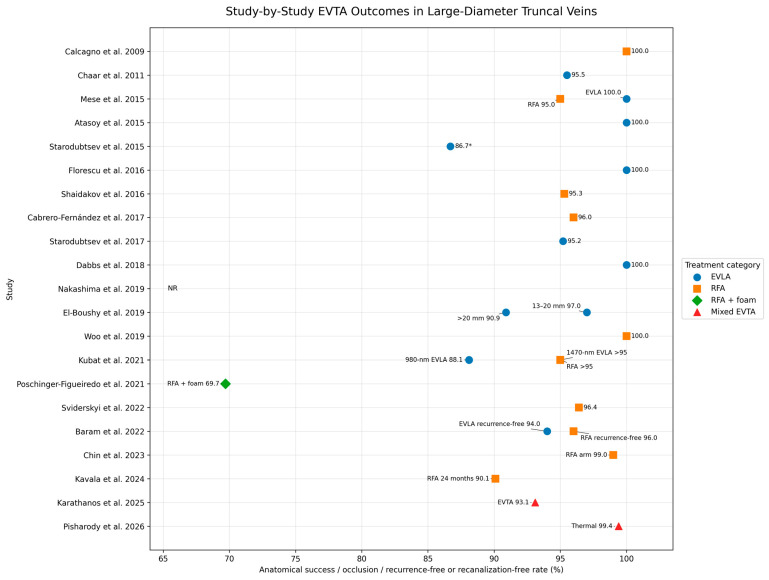
Study-by-study EVTA outcomes in large-diameter truncal veins [4,5,6,7,8,9,10,11,12,13,14,15,16,17,18,19,20,21,22,23,24]. The figure summarizes anatomical success, occlusion, recurrence-free, or recanalization-free rates at the longest available follow-up in studies evaluating EVTA. NR indicates an included EVTA-related study without a single directly plottable exact numeric EVTA outcome. The value for Starodubtsev et al. 2015 [9] is marked with an asterisk because it was derived as 100% minus the reported 13.3% repeat EVLA rate. For values reported as >95%, these are displayed at 95% for plotting purposes only [17]. For Baram et al. 2022 [20], recurrence-free rates were calculated as 100% minus the reported recurrence rates. Abbreviations: EVLA, endovenous laser ablation; EVTA, endovenous thermal ablation; NR, not reported/not directly plottable; RFA, radiofrequency ablation.

Among studies reporting numerical anatomical outcomes for large-diameter EVTA-treated veins, occlusion, anatomical success, recurrence-free, or recanalization-free rates at the longest available follow-up ranged from 69.7% to 100% (Figure 2). The lowest reported long-term occlusion rate was observed in the RFA plus foam sclerotherapy cohort reported by Poschinger-Figueiredo et al. [18]. Several EVLA and RFA studies reported closure rates above 95% [4,5,7,8,13,16]. Karathanos et al. reported 24-month occlusion of 93.1% in GSVs ≥ 12 mm [23]. Pisharody et al. analyzed 16,937 thermal ablation procedures from the Vascular Quality Initiative Varicose Vein Module, including 3163 procedures in large-diameter veins defined as ≥10 mm. In the large-vein cohort, recanalization occurred in 18 procedures (0.6%), corresponding to a recanalization-free rate of 99.4% at a mean follow-up of 174 days [24].

In the EVLA studies included in this review, wavelengths ranged from 810 nm to 1560 nm, with most contemporary series using 1470-nm or 1560-nm systems. Reported anatomical success was generally high, but follow-up duration and outcome definitions varied considerably. Short-wavelength EVLA studies reported closure rates of 95.5–100% at short-term follow-up, although the 980-nm EVLA arm in one comparative study showed a lower 12-month occlusion rate of 88.1% [17]. Long-wavelength EVLA studies reported mostly favorable outcomes, ranging from 90.9% to 100%, with several series using radial or other modified fibers and diameter-adapted energy delivery.

Complication reporting was heterogeneous. Chaar et al. reported two DVTs and eight EHIT or thrombus-extension events after EVLA [6]. Atasoy et al. and Dabbs et al. reported no EHIT events, while Dabbs et al. reported three DVTs [8,13]. Florescu et al. reported one subsegmental pulmonary embolism [4]. No fatal pulmonary embolism was reported in the included EVTA studies.

### 3.4. Ultrasound-Guided Foam Sclerotherapy and Microfoam Ablation

Two primary studies evaluated foam-based treatment in large-diameter truncal veins (Table 4). Barrett et al. investigated microfoam UGFS in veins with junction diameters ≥ 10 mm [25], while Chin et al. compared RFA with microfoam ablation in truncal veins > 8 mm [21]. Follow-up ranged from 14 weeks to a mean of 24.5 months.

Barrett et al. reported that 37.5% of large veins required a second treatment, corresponding to a calculated treatment success without repeat intervention of 62.5% [25]. Chin et al. reported complete truncal vein closure in 94% after microfoam ablation and 99% after RFA at 14 weeks [21]. Common femoral vein thrombus extension occurred in 6.1% after microfoam ablation and 3.0% after RFA.

### 3.5. Cyanoacrylate Closure

Two primary studies evaluated CAC in large-diameter GSVs (Table 5). Kubat et al. compared CAC with HL/S, RFA, and EVLA in GSVs ≥ 10 mm and reported a 12-month CAC occlusion rate of 84.8% [17]. Kavala et al. compared CAC with RFA in GSVs measuring 12–16 mm and followed patients for 24 months [22].

In Kavala et al., full occlusion after CAC was 87.3% at 1 month, 85.9% at 6 months, 81.7% at 12 months, and 77.5% at 24 months. Corresponding RFA closure rates were 100%, 97.2%, 90.1%, and 90.1%, respectively. Early closure rates were significantly higher after RFA at 1 and 6 months, whereas closure rates did not differ significantly at 12 and 24 months. Complication rates were similar between groups, although DVT occurred in 2.8% after CAC and 0% after RFA. Kavala et al. defined anatomical success as complete GSV closure without signs of recanalization [22].

### 3.6. Mechanochemical Ablation

One primary study evaluated MOCA in large-diameter truncal veins (Table 6). Pisharody et al. investigated MOCA using ClariVein in GSVs ≥ 10 mm [26]. At 12 months, occlusion was reported in 88.5% of limbs, corresponding to a recanalization rate of 11.5%. No major adverse events were reported.

### 3.7. High Ligation and Stripping

Three studies evaluated HL/S as a comparator treatment in patients with large-diameter GSVs (Table 7). Definitions of large veins ranged from ≥10 mm to ≥14 mm, and follow-up ranged from 12 to 48 months [10,17,20].

Shaidakov et al. reported a residual venous stump in 6.2% after HL/S and recanalization in 4.7% after RFA at 12 months [10]. Kubat et al. reported recurrence rates of 3.2% after HL/S and 5.5% after 1470-nm EVLA at 12 months [17]. Baram et al. reported Doppler-detected recurrence rates of 6% after HL/S, 6% after EVLA, and 4% after RFA at 48 months [20].

## 4. Discussion

Despite the widespread use of endovenous and surgical treatments for chronic venous disease, evidence specifically addressing large-diameter truncal veins remains limited. The present review identified only 23 primary studies over more than two decades, most of which were observational cohorts or case series. Study design, vein-diameter thresholds, anatomical measurement sites, treatment protocols, follow-up duration, and outcome definitions varied substantially. This heterogeneity limits direct comparison between studies and explains why dedicated randomized controlled trials specifically designed for large-diameter truncal veins remain desirable [2,3].

A major challenge is the absence of a standardized definition of a large-diameter truncal vein. Across the included studies, reported thresholds ranged from >8 mm to >20 mm, although most studies used cutoffs between 10 and 12 mm. The 2022 ESVS guidelines consider truncal veins >12 mm as large-diameter veins [1]. In addition, vein diameter was measured at different anatomical levels, including the junction, proximal thigh, mid-thigh, distal thigh, or maximum treated diameter. Because truncal vein diameter varies along its course, a single measurement may not adequately characterize the treated vein. Future studies should therefore report the anatomical level of measurement, patient position during ultrasound examination, and whether maximum or segment-specific diameters were used.

Athavale et al. and Bontinis et al. provide an important framework for interpreting treatment strategies in this subgroup. Both reviews emphasized the limited and heterogeneous nature of the evidence base [2,3]. In particular, Bontinis et al. demonstrated that increasing GSV diameter was negatively associated with anatomical occlusion after EVTA, suggesting that the relationship between vein diameter and treatment success is continuous rather than based on a single fixed threshold [3]. Therefore, large vein diameter should not be regarded as an absolute contraindication to endovenous treatment, but as a factor that should influence treatment selection and technical strategy.

When modalities are compared according to diameter range and durability, EVTA has the strongest evidence base for large-diameter truncal veins. EVLA and RFA were evaluated across the widest diameter range, including veins > 8 mm, ≥10 mm, ≥12 mm, ≥15 mm, and >20 mm. Most EVTA studies reported anatomical success, occlusion, recurrence-free, or recanalization-free rates above 90% [4,5,6,7,8,9,10,11,12,13,14,15,16,17,19,20,21,22,23,24]. However, in larger veins, particularly those exceeding 12–15 mm, standard energy delivery may be insufficient. Energy delivery should therefore be increased or adapted to vein diameter and anatomical segment. This is supported by segment-based EVLA dosing, including the four-zone dosimetry model for 1940-nm EVLA, in which vein diameters are assessed at multiple anatomical levels and energy delivery is adapted accordingly [27]. The safety and efficacy of 1940-nm EVLA and the broader evidence for EVLA systems emitting at wavelengths >1900 nm have also been reported in previous studies [28,29]. This approach is particularly relevant in the proximal GSV, where diameter and reflux burden are often greatest [27,28,29]. Larger vein diameter may also increase the risk of thrombus extension or EHIT, reinforcing the importance of careful catheter positioning, adequate tumescence, diameter-adapted energy delivery, and post-procedural duplex surveillance [3,6,13,24]. Interpretation of EVLA outcomes in large-diameter truncal veins is limited by substantial technological and procedural heterogeneity. Earlier EVLA studies frequently used lower wavelengths, usually 810–980 nm, with bare-tip fibers, whereas more recent studies have used higher, water-absorbing wavelengths such as 1470, 1560, 1920, or 1940 nm in combination with radial-emitting or other modified fibers [27,28,29]. Higher wavelengths and radial fibers may reduce postoperative pain, bruising, induration, and local inflammatory complications while maintaining high occlusion rates; however, these technological changes are closely linked to differences in LEED, power settings, pullback speed, tumescence, catheter-tip positioning, and adjunctive procedures [27,28,29]. Therefore, outcome differences between EVLA studies should not be attributed to vein diameter alone. The lack of standardized reporting of wavelength, fiber design, dosimetry, stump length, recanalization definitions, and follow-up protocols represents an important limitation of the current evidence base and prevents reliable comparison between EVLA systems in large-diameter veins [29].

Vacuum-assisted laser ablation (VALA) is an emerging modification of EVLA intended to improve the treatment of large, very large, or aneurysmal saphenous veins. The technique is based on the evacuation of residual intraluminal blood during laser ablation, aiming to improve vein-wall contact and reduce blood-mediated heat loss, carbonization, thrombus formation, and post-procedural symptoms. Although VALA is increasingly discussed and used in specialized centers, the available clinical evidence remains very limited and the method cannot yet be considered well-established. The currently available peer-reviewed evidence includes a small retrospective series of ten patients with type I GSV aneurysms close to the junction treated with VALA; at 6 months, aneurysms were absent and clinical outcomes improved in all patients, although two EHIT class 1 events and one EHIT class 2 event were reported [30]. A prospective interventional study comparing standard EVLA with VALA for large GSV or SSV incompetence ≥15 mm is currently registered, with planned enrollment of 184 patients [31]. Therefore, VALA appears technically promising for large or aneurysmal saphenous trunks, but its comparative safety, durability, and optimal thromboprophylaxis strategy require prospective evaluation before firm conclusions can be drawn. For these reasons, these studies were not included in the current analysis.

The evidence for non-thermal non-tumescent techniques in large-diameter truncal veins remains less robust. Foam-based treatment and microfoam ablation were evaluated in veins > 8 mm to ≥10 mm, with outcomes ranging from a calculated treatment success without repeat intervention of 62.5% to short-term closure of 94% [21,25]. CAC and MOCA have also been evaluated in large veins, but the available data remain limited. CAC showed 84.8% occlusion at 12 months in GSVs ≥ 10 mm in Kubat et al. [17], while Kavala et al. reported decreasing full occlusion after CAC in GSVs measuring 12–16 mm, from 87.3% at 1 month to 77.5% at 24 months, compared with 90.1% after RFA at 24 months [22]. Although CAC can therefore be used in selected large-diameter GSVs, its long-term durability appears less predictable than thermal ablation in larger veins. This is consistent with Athavale et al., who highlighted lower CAC occlusion compared with 1470-nm EVLA or RFA in large veins and emphasized the lack of long-term patency and repeat-intervention data [2]. MOCA was evaluated in one large-vein-specific study and achieved 88.5% occlusion at 12 months in GSVs ≥ 10 mm [26]. Based on the current evidence, CAC and MOCA may be best suited to smaller or moderately enlarged truncal veins, whereas their durability in larger veins remains insufficiently established.

HL/S remains relevant as both a comparator and a selective treatment option. In studies including veins ≥ 10–14 mm, reported rates of recurrence, Doppler-detected recurrence, or residual venous stump were low, ranging from 3.2% to 6.2% [10,17,20]. These outcomes, however, were defined heterogeneously and should not be directly compared with duplex-confirmed anatomical occlusion after EVTA. Surgical treatment may still be appropriate in selected cases, particularly in patients with very large, tortuous, aneurysmal, or otherwise catheter-unfavorable anatomy, or when durable endovenous closure is considered unlikely.

Although the present review focused on diffuse large-diameter truncal venous incompetence, focal aneurysmal dilatation of the GSV or SSV represents a related but distinct entity. True saphenous vein aneurysms are rare, and their prevalence is not well-defined. In contrast to diffuse enlargement of an incompetent truncal vein, saphenous vein aneurysms are localized dilatations and may be associated with local thrombus formation, and in exceptional cases, pulmonary embolism. Small series have reported the feasibility of endovenous thermal ablation for incompetent saphenous veins with aneurysmal dilatation close to the junction. Hamann et al. reported EVTA with or without high ligation for saphenous aneurysms close to the junction, while Pavlović et al. reported long-term results of RFA for type I GSV aneurysms [32,33]. However, these studies were small and anatomically distinct from the diffuse large-diameter refluxing trunks evaluated in the present review. Therefore, focal saphenous vein aneurysm studies were excluded from the main analysis and should be interpreted separately from studies of diffuse truncal enlargement.

Overall, the available evidence supports an individualized, anatomy- and diameter-adapted treatment strategy rather than a uniform modality-based approach. Treatment selection should consider truncal vein diameter, anatomical course, tortuosity, junctional morphology, reflux pattern, patient preference, and the expected durability of closure. EVTA, non-thermal techniques, and HL/S may each have a role in selected patients, provided that treatment choice and technical execution are adapted to the anatomical situation. Future studies should use standardized diameter definitions, uniform ultrasound measurement protocols, consistent outcome definitions, and long-term follow-up to allow for meaningful comparison between treatment modalities.

## 5. Limitations

This review has several limitations. First, the available evidence on large-diameter truncal veins remains limited and is dominated by observational studies, retrospective cohorts, registry analyses, and case series. Dedicated randomized controlled trials specifically designed to compare treatment modalities in large-diameter veins were not identified.

Second, substantial heterogeneity was present across the included studies. Definitions of large-diameter veins varied widely, and vein diameter was measured at different anatomical levels. Some studies reported maximum vein diameter, whereas others used junctional, proximal thigh, or segment-specific measurements. These differences limit direct comparison between studies.

Third, outcome reporting was inconsistent. Studies reported anatomical occlusion, technical success, recurrence, residual venous stump, recanalization, reintervention, clinical improvement, or quality-of-life outcomes. These endpoints are not equivalent and should not be interpreted interchangeably. In some studies, success rates were calculated indirectly from reported recurrence or reintervention rates, which may introduce additional uncertainty.

Fourth, follow-up duration varied considerably, ranging from short-term duplex follow-up to 48 months. Therefore, early anatomical success may not reflect long-term durability, particularly for non-thermal techniques such as CAC, MOCA, and microfoam ablation. Finally, complication reporting was incomplete and heterogeneous, precluding a reliable comparison of safety outcomes between modalities.

## 6. Conclusions

Evidence specifically addressing the treatment of large-diameter truncal veins remains limited and heterogeneous. EVTA has the broadest evidence base in this subgroup, whereas evidence for foam-based treatment, CAC, and MOCA is less robust and long-term durability in larger veins remains uncertain. HL/S remains relevant in selected anatomical situations. Treatment should be individualized according to vein diameter, anatomy, tortuosity, reflux pattern, patient preference, and expected durability. Future studies should use standardized diameter definitions, uniform ultrasound measurement protocols, consistent outcome definitions, and long-term follow-up to clarify optimal treatment strategies for large-diameter truncal veins.

## Figures and Tables

**Table 1:** Evidence level of the included primary studies.

Evidence Category	Studies, *n*	Comment
Comparative treatment/protocol study	7	Non-randomized comparisons of EVLA vs. RFA, RFA vs. HL/S, EVLA strategies, RFA vs. CAC, microfoam vs. RFA, or multiple approaches
Diameter-comparison observational cohort	10	Studies comparing predefined large-diameter groups with smaller-vein groups
Prospective observational study	1	Prospective single-arm observational study
Observational case series/technical series	4	Single-arm case series or technical outcome series
Registry/database study	1	Retrospective registry/database analysis
Randomized controlled trial	0	No true RCT specifically designed for large-diameter truncal veins
Total primary studies	23	Included in qualitative synthesis

**Table 2:** Summary of evidence across treatment modalities.

Modality	Studies, *n* *	Treatment Types	Large-Vein Definitions	Follow-Up Range
EVTA	21	EVLA, RFA, or mixed thermal ablation cohorts	>8 mm to >20 mm	3 weeks to 48 months
UGFS/microfoam	2	Foam ultrasound-guided sclerotherapy; polidocanol microfoam ablation	>8 mm to ≥10 mm	14 weeks to 24 months
CAC	2	CAC as standalone treatment or comparator	≥10 mm; 12–16 mm	12 to 24 months
MOCA	1	MOCA/ClariVein	≥10 mm	12 months
HL/S or stripping-based surgery	4	HL/S; EVLA combined with stripping	≥10 mm to ≥14 mm	12 to 48 months

* Modality counts are not mutually exclusive because several studies contributed data to more than one modality. Abbreviations: CAC, cyanoacrylate closure; EVLA, endovenous laser ablation; EVTA, endovenous thermal ablation; HL/S, high ligation and stripping; MOCA, mechanochemical ablation; RFA, radiofrequency ablation; UGFS, ultrasound-guided foam sclerotherapy.

**Table 3:** Studies including endovenous thermal ablation in large-diameter truncal veins.

Study	Design	Treatment/Definition	N and Follow-Up	Main Anatomical Outcome	Reported Complications
Calcagno et al. 2009 [5]	Diameter-comparison cohort	RFA; >12 mm	96 veins >12 mm; 6 months	100% closure in >12 mm group	No DVT/PE reported
Chaar et al. 2011 [6]	Retrospective comparative cohort	EVLA, 810 nm, fiber NR; ≥10 mm	732 patients; 3 weeks	95.5% success	2 DVTs; 8 EHIT/thrombus extensions
Mese et al. 2015 [7]	Comparative observational	EVLA, 1470 nm, radial fiber vs. RFA; >10 mm	120 patients; 6 months	EVLA 100%; RFA 95%	Not reported
Atasoy et al. 2015 [8]	Case series	EVLA, 1470 nm, 600-µm bare-tip fiber; 15–26 mm	44 patients/49 veins; 12 months	100% after repeat treatment where required	No DVT/EHIT reported
Starodubtsev et al. 2015 [9]	Comparative protocol study	EVLA, 1560 nm, fiber NR ± crossectomy/diameter-adapted EVLA; ≥15 mm	88 patients; 12 months	Final obliteration in all groups; 86.7% * primary success in standard-energy group	No DVT/PE reported
Florescu et al. 2016 [4]	Case series	EVLA, 810 nm, fiber NR; 10–20 mm and >20 mm	38 patients; 3 months	100%	1 subsegmental PE
Shaidakov et al. 2016 [10]	Comparative observational	RFA vs. HL/S; ≥14 mm	129 patients; 12 months	RFA 95.3%	Not reported
Cabrero-Fernández et al. 2017 [11]	Prospective diameter-comparison cohort	RFA; ≥12 mm	74 large GSVs; 12 months	96%	Not reported
Starodubtsev et al. 2017 [12]	Prospective observational	EVLA, 1560 nm, fiber NR; >15 mm	210 patients; 6 months	95.2%	Not reported
Dabbs et al. 2018 [13]	Technical/observational series	EVLA, 1470 nm, fiber NR; ≥15 mm	929 patients; 8 weeks	100%	No EHIT; 3 DVTs
Nakashima et al. 2019 [14]	Observational series	EVLA, 1470 nm, ELVeS Radial 2ring fiber + stripping; maximum GSV > 15 mm	42 patients/51 limbs; 3 months	Exact anatomical occlusion not separately reported	Clinical improvement reported
El-Boushy et al. 2019 [15]	Prospective diameter-comparison cohort	EVLA, 1470 nm, Biolitec radial-tip laser catheter; 13–20 mm and >20 mm	259 veins; 12 months	97.0% for 13–20 mm; 90.9% for >20 mm	Not reported
Woo et al. 2019 [16]	Retrospective diameter-comparison cohort	RFA; >12 mm	59 large GSVs; 12 months	100%	No significant difference in complications
Kubat et al. 2021 [17]	Comparative observational	EVLA, 980 nm and 1470 nm, fiber NR /RFA/CAC/HL/S; ≥10 mm	671 patients; 12 months	980-nm EVLA 88.1%; 1470-nm EVLA and RFA > 95%	Not consistently reported
Poschinger-Figueiredo et al. 2021 [18]	Prospective cohort	RFA + foam; ≥13 mm	33 veins; 36 months	69.7%	Not reported
Sviderskyi et al. 2022 [19]	Retrospective diameter-comparison cohort	RFA; >12 mm	282 large GSVs; 12 months	96.3–96.4%	Not reported
Baram et al. 2022 [20]	Prospective non-randomized comparative	EVLA, 1470 nm, fiber/device NR vs. RFA vs. HL/S; ≥10 mm	150 patients; 48 months	EVLA/RFA recurrence-free >90%	1 DVT reported
Chin et al. 2023 [21]	Retrospective comparative cohort	RFA vs. microfoam; >8 mm	132 veins; 14 weeks	RFA 99%; microfoam 94%	CFV thrombus extension: RFA 3.0%, microfoam 6.1%
Kavala et al. 2024 [22]	Retrospective comparative	RFA vs. CAC; GSV 12–16 mm	RFA *n* = 71; 24 months	RFA 90.1% full occlusion	DVT: RFA 0%, CAC 2.8%
Karathanos et al. 2025 [23]	Retrospective diameter-comparison study	EVTA, including 1470-nm EVLA arm, fiber NR; ≥12 mm	87 limbs ≥ 12 mm; 24 months	93.1%	No difference in adverse events
Pisharody et al. 2026 [24]	Registry/database study	Thermal ablation, laser/RFA; EVLA-specific wavelength and fiber NR; ≥10 m	3163 large-vein procedures; mean 174 days	99.4% without recanalization	Hematoma 0.7%; superficial phlebitis 1.5%; DVT 0.9%

* The value represents primary success without repeat EVLA in the standard-energy group, calculated as 100% minus the reported 13.3% repeat EVLA rate [9]. Abbreviations: CAC, cyanoacrylate closure; CFV, common femoral vein; DVT, deep vein thrombosis; EHIT, endothermal heat-induced thrombosis; EVLA, endovenous laser ablation; EVTA, endovenous thermal ablation; GSV, great saphenous vein; HL/S, high ligation and stripping; PE, pulmonary embolism; RFA, radiofrequency ablation; NR, not reported.

**Table 4:** Studies evaluating UGFS/microfoam ablation in large-diameter truncal veins.

Study	Design	Treatment/Definition	N and Follow-Up	Success/Closure	Failure/Complications
Barrett et al. 2004 [25]	Diameter-comparison cohort	Microfoam UGFS; junction diameter ≥ 10 mm	17 large veins; mean 24.5 months	62.5% treatment success without repeat intervention *	37.5% required second treatment; complications not clearly reported
Chin et al. 2023 [21]	Retrospective comparative cohort	RFA vs. microfoam; >8 mm	132 veins; 14 weeks	Microfoam 94%; RFA 99%	Microfoam 6%; RFA 1%; CFV extension: microfoam 6.1%, RFA 3.0%

* Calculated as 100% minus the reported 37.5% second-treatment rate; this reflects treatment success without repeat intervention rather than directly reported duplex-confirmed anatomical closure. Abbreviations: CFV, common femoral vein; RFA, radiofrequency ablation; UGFS, ultrasound-guided foam sclerotherapy.

**Table 5:** Studies evaluating cyanoacrylate closure in large-diameter truncal veins.

Study	Design	Treatment/Definition	N and Follow-Up	Occlusion/Success	Failure/Complications
Kubat et al. 2021 [17]	Comparative observational	CAC vs. HL/S, RFA, EVLA; GSV ≥ 10 mm	671 patients total; 12 months	CAC 84.8%	CAC 15.2%; complications not consistently reported
Kavala et al. 2024 [22]	Retrospective comparative	CAC vs. RFA; GSV 12–16 mm	142 patients; CAC *n* = 71, RFA *n* = 71; 24 months	CAC 77.5%; RFA 90.1% at 24 months	CAC patency 14.1%; DVT: CAC 2.8%, RFA 0%

Abbreviations: CAC, cyanoacrylate closure; DVT, deep vein thrombosis; EVLA, endovenous laser ablation; GSV, great saphenous vein; HL/S, high ligation and stripping; RFA, radiofrequency ablation.

**Table 6:** Study evaluating mechanochemical ablation in large-diameter truncal veins.

Study	Design	Treatment/Definition	N and Follow-Up	Occlusion/Success	Failure/Complications
Pisharody et al. 2024 [26]	Retrospective cohort	MOCA/ClariVein; GSV ≥ 10 mm	104 limbs; 12 months	88.5%	11.5% recanalization; no major adverse events reported

Abbreviations: GSV, great saphenous vein; MOCA, mechanochemical ablation.

**Table 7:** Studies evaluating high ligation and stripping in large-diameter truncal veins.

Study	Design	Treatment/Definition	N and Follow-Up	Success/Freedom From Recurrence	Failure/Recurrence
Shaidakov et al. 2016 [10]	Retrospective comparative cohort	HL/S vs. RFA; GSV ≥ 14 mm	129 patients; 12 months	HL/S 93.8% with no residual venous stump	Residual venous stump after HL/S 6.2%; RFA recanalization 4.7%
Kubat et al. 2021 [17]	Comparative observational	HL/S vs. EVLA/RFA/CAC; GSV ≥ 10 mm	671 patients; 12 months	HL/S 96.8% reported recurrence-free	Reported recurrence: HL/S 3.2%; 1470-nm EVLA 5.5%
Baram et al. 2022 [20]	Prospective non-randomized comparative	HL/S vs. EVLA vs. RFA; GSV ≥ 10 mm	150 patients; 48 months	HL/S 94.0% Doppler recurrence-free	Doppler recurrence: HL/S 6%; EVLA 6%; RFA 4%

Abbreviations: CAC, cyanoacrylate closure; EVLA, endovenous laser ablation; GSV, great saphenous vein; HL/S, high ligation and stripping; RFA, radiofrequency ablation.

## Data Availability

No new data were created or analyzed in this study. Data sharing is not applicable to this article.
